# Oncogenic role of ALX3 in cervical cancer cells through KDM2B-mediated histone demethylation of CDC25A

**DOI:** 10.1186/s12885-021-08552-7

**Published:** 2021-07-16

**Authors:** Jinhong Qi, Li Zhou, Dongqing Li, Jingyuan Yang, He Wang, Huifang Cao, Yunlan Huang, Zhiming Zhang, Linlin Chang, Chenhao Zhu, Juntong Zhan, Yong Yuan

**Affiliations:** 1 Department Two of Gynecologic Oncology, Jilin Cancer Hospital, No. 1018, Huguang Road, Changchun, 130012 Jilin People’s Republic of China; 2 Department of Anesthesiology, Jilin Cancer Hospital, Changchun, 130012 Jilin People’s Republic of China

**Keywords:** ALX3, KDM2B, CDC25A, Cervical cancer; proliferation; cell cycle

## Abstract

**Background:**

Cell division cycle 25A (CDC25A) is a well-recognized regulator of cell cycle progression and is involved in cancer development. This work focused on the function of CDC25A in cervical cancer cell growth and the molecules involved.

**Methods:**

A GEO dataset GSE63514 comprising data of cervical squamous cell carcinoma (CSCC) tissues was used to screen the aberrantly expressed genes in cervical cancer. The CDC25A expression in cancer and normal tissues was predicted in the GEPIA database and that in CSCC and normal cells was determined by RT-qPCR and western blot assays. Downregulation of CDC25A was introduced in CSCC cells to explore its function in cell growth and the cell cycle progression. The potential regulators of CDC25A activity and the possible involved signaling were explored.

**Results:**

CDC25A was predicted to be overexpressed in CSCC, and high expression of CDC25A was observed in CSCC cells. Downregulation of CDC25A in ME180 and C33A cells reduced cell proliferation and blocked cell cycle progression, and it increased cell apoptosis. ALX3 was a positive regulator of CDC25A through transcription promotion. It recruited a histone demethylase, lysine demethylase 2B (KDM2B), to the CDC25A promoter, which enhanced CDC25A expression through demethylation of H3k4me3. Overexpression of ALX3 in cells blocked the inhibitory effects of CDC25A silencing. CDC25A was found as a positive regulator of the PI3K/Akt signaling pathway.

**Conclusion:**

This study suggested that the ALX3 increased CDC25A expression through KDM2B-mediated demethylation of H3K4me3, which induced proliferation and cell cycle progression of cervical cancer cells.

**Supplementary Information:**

The online version contains supplementary material available at 10.1186/s12885-021-08552-7.

## Background

Cervical cancer is the fourth most prevalent gynecological malignancy and the fourth leading cause of cancer mortality in females worldwide [1]. Cervical squamous cell carcinoma (CSCC) represents the most frequent type which accounts for approximately 80% of all cases of cervical cancer, and nearly 90% of these cases are caused by human papillomavirus (HPV) infection [2, 3]. Thanks to the increasing administration of HPV vaccines [4, 5], the incidence rate of cervical cancer is expected to experience a significant decline. However, a substantial increase in cervical cancer morbidity has been seen in China, which may be caused by the inadequate Papanicolaou test screening and less coverage of HPV vaccines [6]. For the infected patients, despite of the progress in diagnostic and therapeutic strategies, the survival rate remains unfavorable [7]. In particular, the overall survival rate of patients at advanced stages was low at approximately 40% even following the conventional chemo-radiotherapies [8]. Therefore, developing novel less-invasive options for cervical cancer treatment is of great significance.

Cancer is believed as a complex product of a progressive series of genetic and epigenetic aberrations. Aberrant transcription activity of genes, in concert with the deregulated epigenetic modifications, is frequently involved in the progression of cancers [9]. In this paper, cell division cycle 25A (CDC25A) was predicted as a candidate oncogene in cervical cancer according to the integrated bioinformatics analyses. CDC25A is a member of the CDC25 phosphatases that govern key transitions between cell cycle phases, which are frequently overexpressed in cancers [10]. CDC25A participates in several biological processes, including cell division, cell cycle progression, DNA replication, cell proliferation, and regulation of cyclin-dependent kinases (CDKs) [11]. In general, it removes inhibitory phosphorylation of CDKs such as CDK2, CDK4 and CDK6 and activates the CDKs that lead to cell cycle progression from the G1 to the S phase [12, 13]. CDC25A has also been reported to be necessary for cervical cancer cell progression [14]. However, the regulatory networks involving CDC25A in cervical cancer development remain largely unknown. The following analyses predicted that ALX3 is a highly potential transcription regulator of CDC25A. An integrated genome-wide analysis by Sun et al. suggested ALX3 as one of the mostly differentially expressed genes (DEGs) in hepatocellular carcinoma [15]. However, to the best of our knowledge, there is limited evidence concerning the role of ALX3, as a transcription factor, in human cancer progression. Therefore, we planned to validate the possible links between ALX3 and CDC25A and their functions in cervical cancer.

Histone methylation is one of the key epigenetic events closely linked to cancer occurrence. Histone H3 trimethylation at lysine 4 (H3K4me3) has been summarized to modulate the expression of tumor-related genes and to be altered during cancer progression [16]. Our bioinformatics analysis indicated that there are H3K4 methylation sites on the histone of CDC25A promoter. We speculated that there might be a specific histone methyltransferase/demethylase that mediates CDC25A transcription through the histone modification of H3K4. Histone lysine demethylases (KDMs) including KDM1A, KDM2B, and KDM5A/B/C/D are well-known to bind to the H3K4me3 sites [9]. Among them, KDM2B was predicted to have a high correlation with CDC25A. Interestingly, knockdown of KDM2B has once been reported to suppress proliferation of cervical cancer cells [17]. Whether KDM2B regulates CDC25A expression through modifying H3K4me3 aroused our attention. Taken together, this study was performed to validate the potential binding relationship between ALX3 and CDC25A promoter and the potential involvements of KDM2B. Altered expression of CDC25A and ALX3 was introduced in vitro and in vivo to access their functions in cervical cancer development.

## Methods

### Bioinformatics analyses

A cervical cancer dataset GSE63514 was obtained from the Gene Expression Omnibus (GEO) database. An R Limma Package (http://www.bioconductor.org/packages/release/bioc/html/limma.html) was applied to analyze the DEGs, and the heatmaps were produced using the R pheatmap package. Difference in CDC25A expression in The Cancer Genome Atlas (TCGA)-CESC (cervical squamous cell carcinoma and endocervical adenocarcinoma tissues) and GTEx-Cervix (normal tissues) was analyzed on the Gene Expression Profiling Interactive Analysis (GEPIA) (http://gepia.cancer-pku.cn/index.html). The binding relationship between CDC25A promoter and the transcription factors was predicted on JASPAR (http://jaspar.genereg.net/). The histone methylation level at the CDC25A promoter was predicted using the UCSC Browser (http://bio.lundberg.gu.se/courses/vt13/ucsc.html). The correlations between CDC25A and ASCL1, ARNT, ALX3, KDM1A, KDM2B or KDM5A/B/C/D in TCGA-CESC were predicted using the GEPIA system as well.

### Cell culture

CSCC cell lines (CaSki, ME180, C33A and Hela) and a primary cervix epithelial cell line (PCS-480) purchased from American Type Culture Collection (ATCC, Manassas, VA, USA) were confirmed free of mycoplasma contamination through an analysis in the short-sequence tandem repeat region. Cells were cultured in Dulbecco’s modified Eagle’s medium (DMEM) supplemented with 100 μg/mL penicillin/streptomycin and 10% fetal bovine serum (Nobleryder Technology Co., Ltd., Beijing, China).

### Cell transfection

PcDNA3.1 vector containing short-hairpin RNA (shRNA) of CDC25A (sh-CDC25A), overexpressing vector of ALX3 (oe-ALX3) or the corresponding negative controls (sh-NC or oe-NC) were synthesized by Genechem Co., Ltd. (Shanghai, China). ME180 and C33A cells were seeded in 6-well plates at 1 × 10^5^ cells per well. Once reaching a 70–80% confluence, the cells were transfected with pcDNA3.1 vector (a final concentration of 50 nM) using a Lipofectamine 2000 kit (Invitrogen, Carlsbad, CA, USA). After 48 h, the transfection efficacy was determined using reverse transcription-quantitative polymerase chain reaction (RT-qPCR).

### RT-qPCR

After transfection, total RNA from ME180 and C33A cells was collected using the TRIzol Reagent (Takara Biotechnology Ltd., Dalian, China). The RNA was first reverse-transcribed to cDNA using a PrimeScript™ RTreagent Kit (Takara). Then, qPCR was determined using a SYBR Premix Ex Taq Kit (Takara) on a CFX96 touch q-PCR system (Bio-Rad, Inc., Hercules, CA, USA) according to the manufacturer’s instructions. The primer sequences are presented in Table 1, where GAPDH was used as the internal reference. Relative mRNA expression was determined by the 2^-ΔΔct^ method.

**Table 1:** Primer sequences for RT-qPCR

Gene	Primer sequence (5′-3′)
CDC25A	F: TCTGGACAGCTCCTCTCGTCAT
	R: ACTTCCAGGTGGAGACTCCTCT
ALX3	F: GGAGAAGGTCTTCCAGAAAACCC
	R: ACTTGGCTCTGCGGTTCTGGAA
KDM2B	F: CATGGAGTGCTCCATCTGCAATG
	R: ACTTCGGACACTCCCAGCAGTT
GAPDH	F: GTCTCCTCTGACTTCAACAGCG
	R: ACCACCCTGTTGCTGTAGCCAA

*RT-qPCR* reverse transcription-quantitative polymerase chain reaction, *CDC25A* cell division cycle 25A, *KDM2B* lysine demethylase 2B, *GAPDH* glyceraldehyde-3-phosphate dehydrogenase, *F* forward, *R* reverse

### Western blot analysis

Total protein from cells was collected from the lysates in radio-immunoprecipitation assay (RIPA) cell lysis buffer. The protein concentration was determined using a bicinchoninic acid (BCA) kit (Thermo Fisher). Next, an equal volume of protein sample (100 μg) was separated on 5, 8% or 12% sodium dodecyl sulfate-polyacrylamide gel electrophoresis and transferred on polyvinylidene fluoride membranes (Millipore, Billerica, MA, USA). The membranes were blocked in 5% bovine serum albumin (BSA) and then incubated with the primary antibodies against CDC25A (SAB4503736, Merck KGaA, Darmstadt, Germany), ALX3 (ab64985, Abcam Inc., Cambridge, MA, USA), KDM2B (17–10,264, Merck), H3K4me3 (ab8580, Abcam) and GAPDH (ab8245, Abcam) at 4 °C overnight. Then, the membranes were stained with the secondary antibody goat anti-rabbit IgG (AP307-P, Merck) at 20 °C for 2 h. The protein blots were developed by enhanced chemiluminescence (Pierce, Waltham, MA, USA) and photographed using the Bio-Rad imaging system. The grey value of the clear protein bands was analyzed.

### Flow cytometry

Flow cytometry was used to measure cell cycle progression and cell apoptosis. For cell cycle measurement, cells were washed in phosphate-buffered saline (PBS), fixed in cold ethanol (70%), and then centrifuged at 2000 rpm for 5 min with the supernatant discarded. The cells were stained with propidium iodide (PI) (Invitrogen) and analyzed on a flow cytometer (BD Biosciences, San Jose, CA, USA). To each sample, 25,000 cells were used, and the results were analyzed using the FlowJo software (BD Biosciences). For apoptosis detection, cells were seeded at 2 × 10^5^ cells per well in 6-well plates. The cells were washed in PBS and resuspended in 1× binding buffer. Then, 100 μL cell suspension (1 × 10^5^ cells) was loaded in 5-mL tubes and incubated with 5 μL Annexin V-fluorescein isothiocyanate (FITC) and 5 μL PI at room temperature (25 °C) in the dark for 15 min, respectively. Next, each tube was further added with 400 μL 1× binding buffer, and the samples were analyzed on the flow cytometer within 1 h. The green fluorescence of Annexin V-FITC was determined at 530 nm while the red fluorescence of PI was determined at 585 nm. The results were analyzed by FlowJo.

### Colony formation assay

The transfected cells were placed in 6-well plates at 1000 cells per well and incubated at 37 °C for 2 weeks. Thereafter, the colonies were fixed in 4% paraformaldehyde and stained with 0.4% crystal violet for 30 min. The number of cell colonies (over 50 cells) was counted under a microscope (Olympus Optical Co., Ltd., Tokyo, Japan).

### 5-ethynyl-2′-deoxyuridine (EdU) labeling assay

Cells were sorted in 24-well plates at a density of 5 × 10^3^ cells per well. Then, the EdU solution was added into the plates for 2 h of warm incubation. Thereafter, the cells were fixed in 4% paraformaldehyde for 30 min and incubated with glycine solution for 8 min. After that, the cells were washed in PBS containing 0.5% Triton X-100, reacted in Apollo® reaction solution in the dark for 30 min, and incubated with Hoechst 33342 solution (C1022, Beyotime Biotechnology Co., Ltd., Shanghai, China) at 25 °C for 20 min. The labeling was observed under the microscope at a × 400 magnification, where the proliferative cells (EdU-positive) were labeled in red while the total cells (Hoechst 33342-postive) were stained in blue. The proliferation rate was determined as follows: proliferation rate = number of proliferative cells/total cells × 100%.

### Terminal deoxynucleotidyl transferase (TdT)-mediated dUTP nick end labeling (TUNEL)

Apoptotic cells were labeled using an in-situ cell apoptosis detection kit (Roche, Penzberg, Upper Bavaria, Germany) according to the kit’s instructions. The nuclei were stained by 4′, 6-diamidino-2-phenylindole (DAPI). The images were taken under an inverted confocal microscope, and the TUNEL-positive cells were quantified using the Image J software.

### Xenograft tumors in nude mice

Twenty female NOD/SCID nude mice (6 weeks old) purchased from SLAC Laboratory Animal Co., Ltd. (Shanghai, China) were used for in vivo experiments. All animal protocols were approved by the Animal Care and Ethics Committee of Jilin Cancer Hospital. Great efforts were made to reduce the pain of animals. In brief, ME180 or C33A cells stably transfected with sh-CDC25A and sh-NC (2 × 10^6^ cells/mL) were subcutaneously injectd into mice through at the flank. The volume (V) of xenograft tumors was measured using a vernier caliper every 5 d, which was calculated as follows: V = L × W^2^/2, where ‘L’ indicates the length and ‘W’ indicates the width. The animals were euthanized on the 35th d through intraperitoneal injection of pentobarbital sodium (150 mg/kg). The tumor tissues were collected and weighed, and preserved in liquid nitrogen for further analysis.

### Immunohistochemical (IHC) staining

The tumor tissues were cut into sections, rehydrated, and blocked in 5% goat serum for 1 h. The sections were incubated with primary antibodies against Ki-67 (#9449, 1:400, Cell Signaling Technologies (CST), Beverly, MA, USA) at 4 °C overnight and then incubated with SignalStain® Boost IHC Reagent (#8125, CST) at 25 °C for 30 min. Next, the sections were stained using a SignalStain® DAB substrate kit (#81059, CST). The staining was observed under a microscope.

### Chromatin immunoprecipitation (ChIP)-qPCR

A MAGnify ChIP System (Invitrogen) was used according to the manufacturer’s instructions for ChIP-qPCR [18]. In brief, cells were collected and fixed, and the chromatin was crosslinked, extracted and cleaved, and then incubated with anti-ALX3 (ab64985, Abcam) for immunoprecipitation. The primer sequence used was as follows: hsa-CDC25A, GGCTGCGTGTGGCTCATCT (sense) and AGGAAGTGAGACAGAAGCTGGG (anti-sense), and the data were normalized to Input.

### Dual-luciferase reporter gene assay

A dual-luciferase reporter gene assay kit (Promega Corp., Madison, Wisconsin, USA) was used for luciferase assay. In brief, oe-ALX3 vector at different doses was co-transfected with 200 ng pGL3 reporter vector containing CDC25A promoter into 293 T cells. After 48 h, the firefly luciferase activity was determined, which was normalized to the activity of Renilla luciferase (pRL-TK, Promega).

### Co-immunoprecipitation (Co-IP)

The binding relationship between ALX and KDM2B was validated using the Co-IP assay as previously described [19]. C33A or ME180 cells were detached and passaged, and sorted in 6-well plates for normal culture. When the cell confluence reached 70–80%, eukaryotic expression vector of ALX3 or KDM2B was transfected into cells. After 48 h of incubation, the cells were lysed in 500 μL RIPA cell lysis buffer on ice, and then the cells were collected and centrifuged at 12,000 rpm at 4 °C for 15 min to collect the supernatant. The IP assay was performed using Protein G plus-Arogose and anti-Flag Affinity Gel, respectively, and then the expression of ALX3 and KDM2B in the precipitates was examined using western blot analysis as aforementioned.

### Subcellular localization of ALX3 and KDM2B by double-labeled immunofluorescence 

The C33A or ME180 cells were sorted in 6-well plates and fixed in 1 mL 4% paraformaldehyde for 30 min. The cells were washed in PBS for three times and added with 0.1%TritonX-100 and then allowed to stand at 25 ***°***C for 15 min. Next, the cells were incubated with mouse anti-human ALX3 and rabbit anti-human KDM2B at 4 °C overnight, and then with FITC-labeled goat anti-mouse IgG or Alexa Fluoro 488-labeld goat anti-rabbit IgG at 25 ***°***C for 2 h. Then, the nuclei were counterstained with DAPI. The staining was observed under a laser confocal microscope (Olympus).

### Statistical analysis

Measurement data were presented as the mean ± standard deviation (SD) from three independent experiments. All cellular experiments were performed in triplicates and in three duplicated wells. Differences were analyzed by the *t* test (two groups) and one-way or two-way analysis of variance (ANOVA, over two groups), and Tukey’s multiple test was used for post hoc test after ANOVA. SPSS21.0 (IBM Corp. Armonk, NY, USA) was used for data analysis. *p < 0.05 indicates statistical significance.

## Results

### CDC25A is highly expressed in cervical cancer samples

**Fig. 1:**
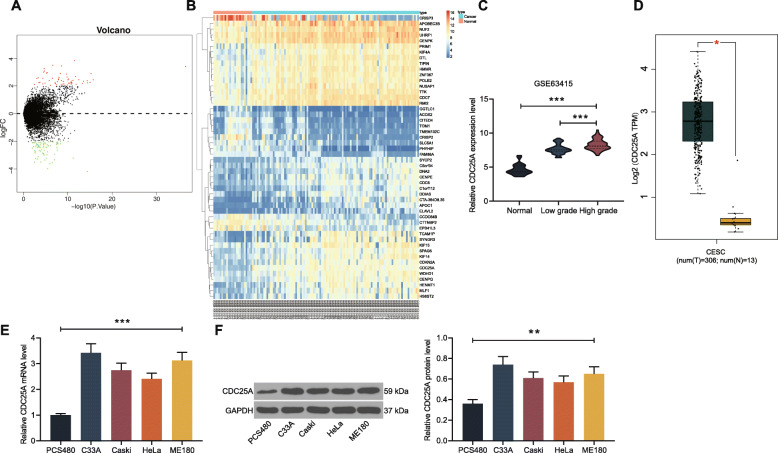
CDC25A is highly expressed in cervical cancer. **A** volcano plots representing the DEGs in the GEO GS63514 dataset; **B** a heatmap for top 50 DEGs between 24 cases of normal cervical epithelial tissues and 104 cases of CSCC tissues; **C** relevance between CDC25A expression and CSCC grade according to the data in the GSE63514 chip; **D** CDC25A expression in TCGA-CESC (data of CESC tissues) and GTEx-Cervix (data of normal cervical tissues) in the GEPIA database; **E**-**F** mRNA (**E**) and protein (**F**) expression of CDC25A in CSCC cell lines (C33A, Caski, HeLa and ME180) and normal cervical epithelial cells (PCS480) determined by RT-qPCR and western blot analysis. Data were presented as the mean ± SD from three independent experiments. In panel **D**, data were analyzed by the unpaired *t* test, while in panels **C**, **E** and **F**, data were analyzed by one-way ANOVA followed by Tukey’s multiple comparison test. In panel **C**, Low grade/High grade vs. Normal, ***p < 0.001; Low grade vs. High grade, ***p < 0.001; in panel **D**, tumor tissues vs normal tissues, *p* < 0.05; in panels E and F, C33A/Caski/HeLa/ME180 vs. PCS480, **p < 0.01, ***p < 0.001. All cellular experiments were performed in triplicates and three duplicated wells were set

First, the GSE63514 dataset, which comprises data of 24 cases of normal cervical epithelial tissues and 104 cases of CSCC tissues, was obtained from the GEO database and analyzed using the R Limma package. Consequently, a total of 134 differentially expressed mRNAs were screened out (Fig. 1a-b) (Table 2). It was found that CDC25A was highly expressed in the cancer tissues and positively correlated with the tumor staging (Fig. 1c). To further validate the correlation between CDC25A and cervical cancer progression, we further explored the CDC25A expression in TCGA-CESC (data of CESC tissues) and GTEx-Cervix (data of normal cervical tissues) in the GEPIA database. It was indicated that CDC25A was highly expressed in CESC tissues compared to that in normal tissues (Fig. 1d). Thereafter, we detected CDC25A expression in CSCC cell lines (C33A, Caski, HeLa and ME180) and in PCS480 cells. Likewise, increased expression of CDC25A was found in all CSCC cell lines (Fig. 1e-f). These results preliminarily suggested that CDC25A might play important function in cervical cancer progression.

**Table 2:** DEGs in the GSE63514 dataset

Gene Symbol	logFC	adj.*p*.Val	Gene Symbol	logFC	adj.*p*.Val
CDC25A	3.41	5.46E-33	KIF14	2.44	2.76E-08
CDC7	2.44	1.90E-12	TTK	2.07	3.27E-08
DTL	2.88	4.02E-12	DDIAS	2.64	3.71E-08
RMI2	2.25	5.17E-12	HENMT1	2.86	4.81E-08
CRISP2	−4.18	1.29E-11	CRISP3	−5.67	1.14E-07
UHRF1	2.23	1.29E-11	C1orf112	2.15	1.31E-07
CENPK	2.39	1.29E-11	APOBEC3B	2.31	1.61E-07
WDHD1	2.34	6.56E-11	DNA2	2.13	2.74E-07
ZNF367	2.18	1.14E-10	SYNGR3	3.44	3.42E-07
POLE2	2.45	5.03E-10	KIF15	2.39	3.67E-07
TIPIN	2.12	1.36E-09	NUSAP1	2.06	4.16E-07
CDKN2A	2.68	1.56E-09	CTTNBP2	−2.17	5.04E-07
TCAM1P	3.83	2.27E-09	TOM1	−2.02	5.19E-07
PHYHIP	−3.2	3.95E-09	CTA-384D8.35	2.89	5.58E-07
SPAG5	2.4	4.49E-09	MLF1	2.62	9.82E-07
C5orf34	2.2	5.79E-09	CENPE	2.5	1.18E-06
CENPQ	2.24	5.91E-09	HS6ST2	2.44	1.23E-06
HMMR	2.13	7.29E-09	CCDC64B	−2.19	1.61E-06
CDC6	2.34	7.64E-09	SLC5A1	−2.12	1.65E-06
NUF2	2.25	7.64E-09	FAM86A	−2.13	1.73E-06
KIF4A	2.19	1.28E-08	APOC1	2.31	1.73E-06
PRIM1	2.5	2.06E-08	GGTLC1	−2.05	1.73E-06
SYCP2	2.2	2.26E-08	CITED4	−2.63	1.95E-06
ACOX2	−2.02	2.54E-08	EPB41L3	−2.43	2.29E-06

*DEGs* differentially expressed genes

### Knockdown of CDC25A suppresses proliferation but promotes cell cycle arrest and apoptosis of cervical cancer cells

**Fig. 2:**
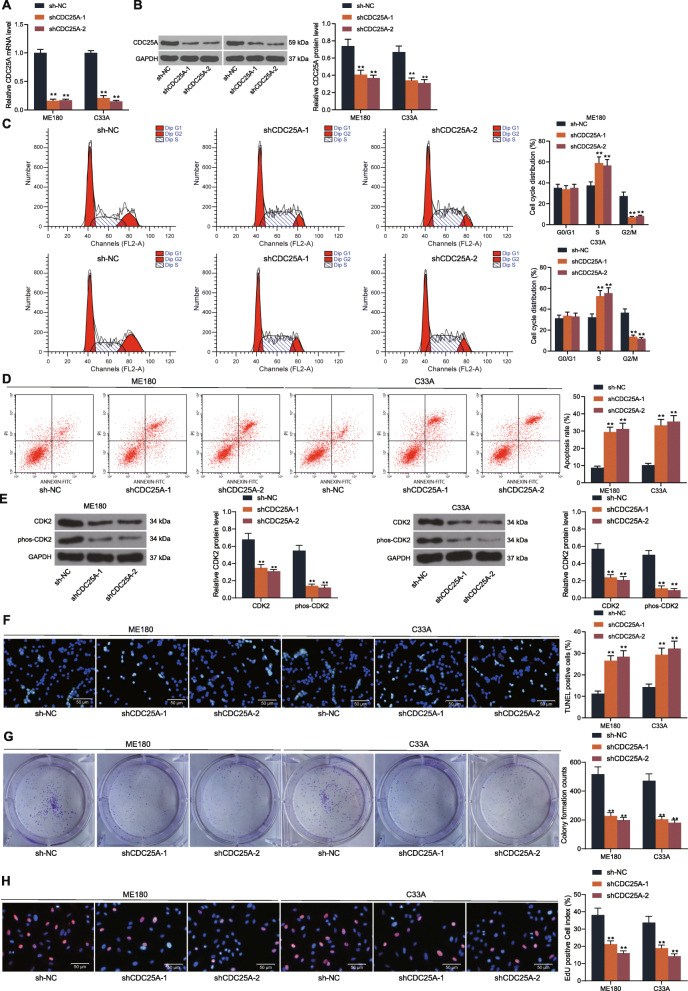
Knockdown of CDC25A suppresses proliferation but promotes cell cycle arrest and apoptosis of CSCC cells. **A**-**B** two shRNAs were administrated in ME180 and C33A cells, after which the CDC25A expression in cells was determined by RT-qPCR and western blot analysis, respectively; **C**-**D** cell cycle progression and apoptosis of ME180 and C33A cells determine by flow cytometry; **E** phosphorylation of CDK2 in ME180 and C33A cells examined by western blot analysis; **F** number of apoptotic cells determined by TUNEL; **G**-**H** proliferation of cells determined by colony formation and EdU labeling assays. Data were presented as the mean ± SD from three independent experiments. Data were analyzed by two-way ANOVA followed by Tukey’s multiple comparison test. In all panels, **p < 0.01 vs. sh-NC. All cellular experiments were performed in triplicates and three duplicated wells were set

To confirm the role of CDC25A in cervical cancer, two shRNAs of CDC25A (sh-CDC25A-1 and sh-CDC25A-2) were administrated into ME180 and C33A cells having the highest CDC25A expression. The successful transfection was confirmed by RT-qPCR and western blot analysis (Fig. 2a-b). CDC25A was a necessary protein for cell cycle progression. Here, after CDC25A knockdown, the cell cycle of ME180 and C33A cells was arrested at the S phase (Fig. 2c). The apoptosis of ME180 and C33A cells was increased (Fig. 2d). In addition, it was found that the inhibitory phosphorylation of CDK2 in ME180 and C33A cells was significantly increased by sh-CDC25A (Fig. 2e). Namely, downregulation of CDC25A reduced CDK2 activity in cells. Likewise, the TUNEL results suggested that the number of TUNEL-positive cells was increased upon CDC25A silencing (Fig. 2f). In addition, the proliferation of cells, according to the colony formation and EdU labeling assays, was suppressed after CDC25A knockdown (Fig. 2g-h). Moreover, the relevance of CDC25A to the growth of PCS480 cells was concerned as well. It was found that overexpression of CDC25A led to a significant increase in the EdU-positive rate as well as number of cell colonies in PCS480 cells (Supplementary Fig. S1A-D). Collectively, these results indicated that CDC25A plays an oncogenic role in cervical cancer progression.

### Knockdown of CDC25A suppresses growth of xenograft tumor in mice

**Fig. 3:**
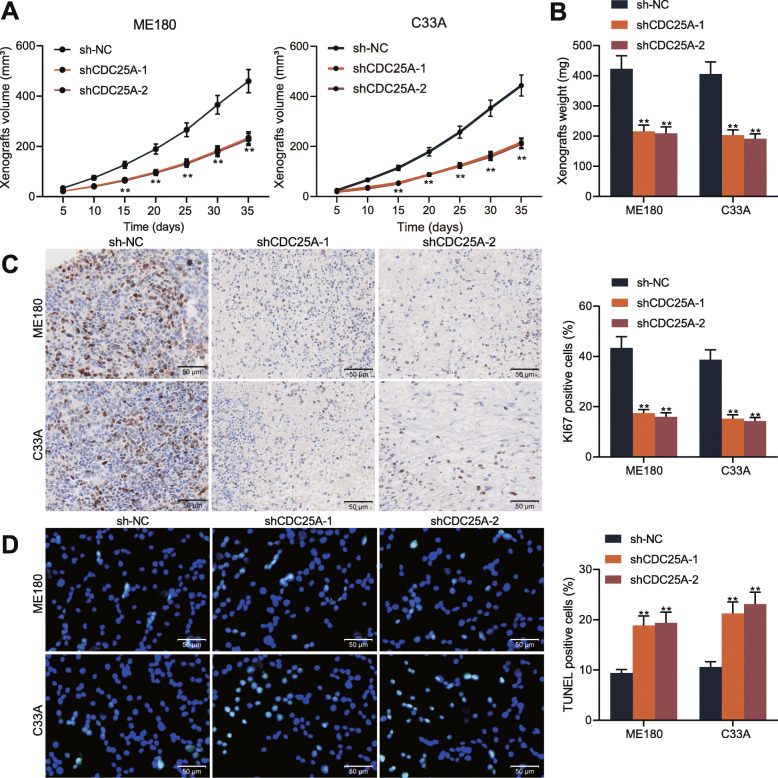
Knockdown of CDC25A suppresses growth of xenograft tumor in mice. **A** ME180 and C33A cells with stable transfection of sh-CDC25A or sh-NC were subcutaneous injection into the flank of the NOD/SCID mice, and then the volume of xenograft tumors was evaluated every 5 d; **B** weight of xenograft tumors; **C** expression of Ki-67 in xenograft tumors determined by IHC staining; **D** number of apoptotic cells in xenograft tumors determined by TUNEL assay. *N* = 5 in each group. Data were presented as the mean ± SD from three independent experiments. Data were analyzed by two-way ANOVA followed by Tukey’s multiple comparison test. In all panels, **p < 0.01 vs. sh-NC

The function of CDC25A in cervical cancer growth in vivo was further explored. ME180 and C33A cells stably transfected with sh-CDC25A or sh-NC were subcutaneously injected into NOD/SCID mice at the flank site. The growth rate of the xenograft tumor was evaluated by the tumor volume changes in every 5 d. It was found that downregulation of CDC25A in either ME180 or C33A cells led to a reduced growth rate in mice (Fig. 3a). On the 35th d after animal euthanasia, the weight of tumors was also reduced after CDC25A knockdown (Fig. 3b). The collected tumors were sectioned for IHC staining. The staining intensity of Ki-67, an important marker for tumor proliferation, was significantly reduced when CDC25A was downregulated (Fig. 3c). In line with the findings in vivo, downregulation of CDC25A led to an increase in the number of apoptotic cells in xenograft tumors (Fig. 3d).

### CDC25A is activated by the transcription factor ALX3

**Fig. 4:**
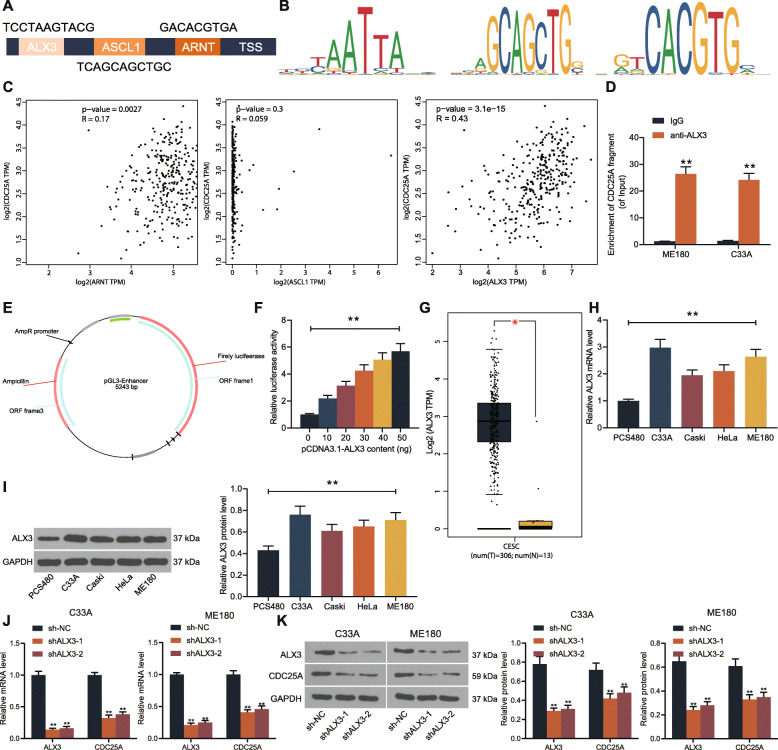
CDC25A is activated by the transcription factor ALX3. **A** potential transcription factors that may bind to the promoter region of CDC25A predicted on JASPAR; **B** conservative binding sites of ASCL1, ARNT and ALX3; **C** correlations between CDC25A expression and ASCL1, ARNT or ALX3 predicted on GEPIA database; **D** binding relationship between ALX3 and the CDC25A promoter validated by the ChIP-qPCR; **E** a diagram for the constructed pGL3-Enhancer-promoter; **F** binding relationship between ALX3 and the CDC25A promoter validated through a luciferase reporter gene assay; **G** ALX3 expression in TCGA-CESC in the GEPIA database; **H**-**I**, mRNA (**H**) and protein (**I**) expression of ALX3 in CSCC cell lines (C33A, Caski, HeLa and ME180) and in normal cervical epithelial cells (PCS480) determined by RT-qPCR and western blot analysis, respectively; **J**-**K** mRNA (**J**) and protein (**K**) expression of ALX3 and CDC25A in ME180 and C33A cells after ALX3 shRNA transfection examined by RT-qPCR and western bot analysis, respectively. Data were presented as the mean ± SD from three independent experiments. Data were analyzed by one-way (**F**, **H**, **I**, **J** and **K**) or two-way (**D**) ANOVA followed by Tukey’s multiple comparison test. In panel **D**, **p < 0.01 vs. IgG; in panel **F**, **p < 0.01 vs. 0 ng pCDNA3.1-ALX3; in panels **H** and **I**, **p < 0.01 vs. PCS-480 cells; in panels **J** and **K**, **p < 0.01 vs. sh-NC. All cellular experiments were performed in triplicates and three duplicated wells were set

To explore the potential upstream regulator of CDC25A, we first predicted the promoter region of CDC25A on the Ensenbl Genome Browser (http://www.ensembl.org/index.html), and then the candidate transcription factors could bind to the promoter region of CDC25A were predicted on JASPAR (http://jaspar.genereg.net/). Three transcription factors ASCL1, ARNT and ALX4 were predicted to possibly bind to CDC25A promoter (Fig. 4a-b). Thereafter, the correlations between CDC25A and ASCL1, ARNT or ALX3 in CESC were predicted on GEPIA. Only ALX3 presented an over 0.35 correlation coefficient with CDC25A (Fig. 4c). To further validate the binding relationship between ALX3 and CDC25A promoter, a ChIP-qPCR was performed using anti-ALX3. An abundance of CDC25A promoter sequence was found in the complexes pulled down by anti-ALX3 compared to anti-IgG (Fig. 4d). In addition, the luciferase reporter vector containing the sequence of CDC25A promoter was co-transfected with oe-ALX3 into 293 T cells for luciferase assay. It was found that the luciferase activity of cells was increased following the increased doses of oe-ALX3 (Fig. 4e-f). In addition, the expression of ALX3 was indicated to be increased in the TCGA-CESC database (Fig. 4g). Then, the ALX3 expression was determined in the CSCC cell lines (C33A, Caski, HeLa and ME180) and in PCS480 cells. Both the mRNA (Fig. 4h) and protein (Fig. 4i) expression of ALX3 was found to be increased in the cancer cell lines. To further validate the regulation of ALX3 on CDC25A expression, shRNA silencing of ALX3 was introduced in ME180 and C33A cells (Fig. 4j-k).

### Overexpression of ALX3 blocks the inhibition of sh-CDC25A on cervical cancer cells

**Fig. 5:**
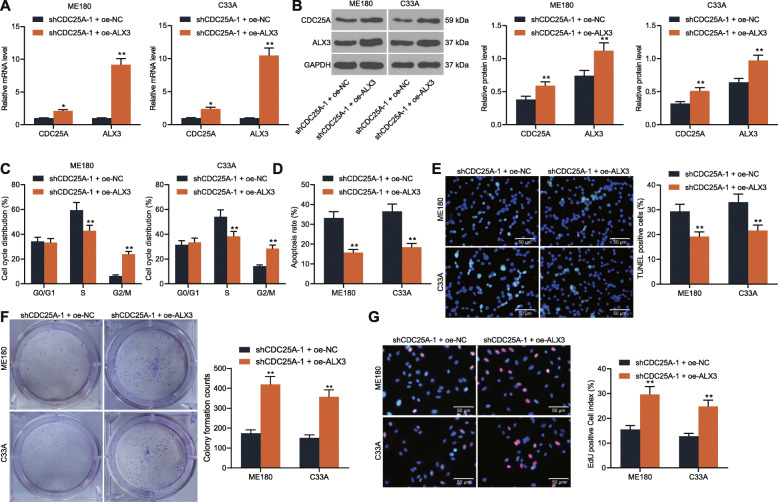
Overexpression of ALX3 blocks the inhibition of sh-CDC25A on CSCC cells. **A**-**B** transfection efficacy of oe-ALX3 in ME180 and C33A cells determined by RT-qPCR (**A**) and western blot analysis (**B**); **C**-**D** cell cycle progression (**C**) and apoptosis (**D**) of ME180 and C33A cells determined by flow cytometry; **E** number of apoptotic cells determined by TUNEL assay; **F**-**G** proliferation ability of cells determined by colony formation (**F**) and EdU labeling (**G**) assays. Data were presented as the mean ± SD from three independent experiments. Data were analyzed by two-way ANOVA followed by Tukey’s multiple comparison test. **p < 0.01 vs. shCDC25A-1 + oe-NC. All cellular experiments were performed in triplicates and three duplicated wells were set

To confirm the role of ALX3 and its interaction with CDC25A onin cervical cancer development, the ME180 and C33A cells with stable downregulation of CDC25A were further administrated with oe-ALX3, and the successful transfection was validated by RT-qPCR and western blot analysis (Fig. 5a-b). Then, it was found that the cell cycle arrest in G2/M phases in both ME180 and C33A cells was notable reduced (Fig. 5c). Still, the increased apoptosis in cells induced by sh-CDC25A was decreased on further ALX3 overexpression (Fig. 5d-e). In addition, the suppressed viability and proliferation of cells was recovered following oe-ALX3 administration (Fig. 5f-g). These results, collectively, suggested that ALX3 positively regulates CDC25A expression to promote cervical cancer cell growth and the cell cycle progression. The function of ALX3 in the growth of PCS480 cells was examined as well. It was found that the expression of CDC25A in PSC480 cells was increased upon ALX3 upregulation. In this setting, the EdU-positive and the number of cell colonies in PSC480 cells were significantly enhanced (Supplementary Fig. S2A-D).

### ALX3 recruits KDM2B to the promoter region of CDC25A to strengthen the CDC25A transcription through demethylation of H3K4me3

**Fig. 6:**
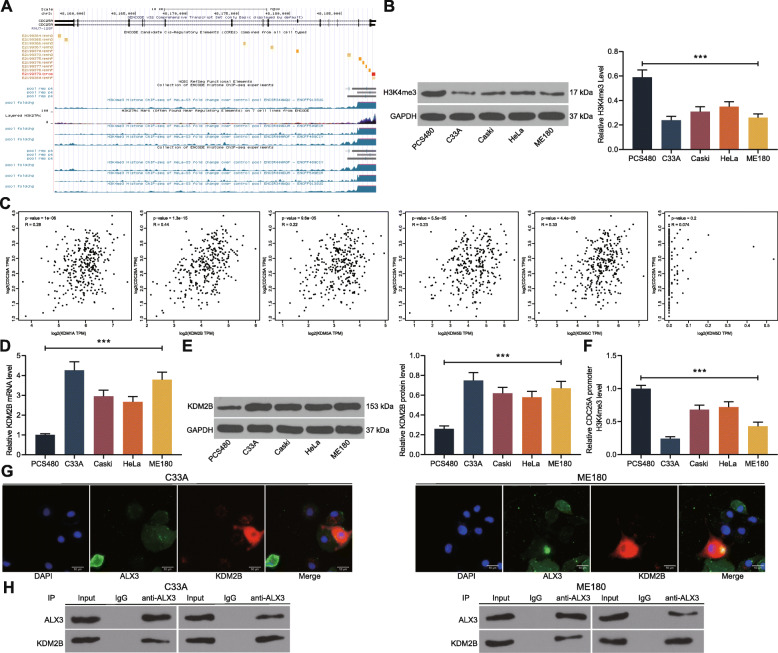
ALX3 recruits KDM2B to the promoter region of CDC25A to strengthen the CDC25A transcription through demethylation of H3K4me3. **A** methylation modification of the CDC25A histone promoter in cervical cancer cells predicted on UCSC browser; **B** expression of H3K4me3 in CSCC cells (C33A, Caski, HeLa and ME180) and in PCS480 cells determined using western blot analysis; **C** correlations between CDC25A and KDM1A, KDM2B, KDM5A/B/C/D predicted on the TCGA-CESC database; **D**-**E**, mRNA (**D**) and protein (**E**) expression of KDM2B in CSCC cells and in PCS480 cells determined by RT-qPCR and western blot analysis, respectively; **F** H3K4me3 expression in CDC25A promoter region in CSCC cells and in PCS480 cells determined by the ChIP-qPCR assays; **G** subcellular localization of ALX3 and KDM2B in ME180 and C33A cells examined by double-labeled immunofluorescence staining; **H** binding relationship between KDM2B and ALX3 in ME180 and C33A cells examined by the Co-IP assay. Data were presented as the mean ± SD from three independent experiments. Data were analyzed by two-way ANOVA followed by Tukey’s multiple comparison test. In panels **B**, **D**, **E** and **F**, ***p < 0.01 vs. PCS480. All cellular experiments were performed in triplicates and three duplicated wells were set

We further predicted the histone modification of CDC25A promoter on the UCSC browser. It was found that the CDC25A promoter histone had significant H3K4 methylation (Fig. 6a). Interestingly, a recent study by Jian Y et al. suggested that downregulation of a histone methyltransferase SMYD3 in ovarian cancer cells decreased the methylation level of histones such as H3K4 and H4K20, which led to increased expression of several genes including CDC25A [20]. This attracted out interests to explore whether ALX3 recruits a certain histone methyltransferase/demethylase to regulate the transcription activity of CDC25A. First, we determined the activity of H3K4me3 in all cell lines. It was found that the level of H3K4me3 was decreased in the CSCC cells (C33A, Caski, HeLa and ME180) compared to that in PCS480 cells (Fig. 6b). As discussed above, several histone demethylases including KDM1A, KDM2B and KDM5A/B/C/D may bind to the H3K4me3 sites to induce its demethylation. Among the histone demethylases, according to the TCGA-CESC database again, only KDM2B showed an over 0.35 correlation coefficient with CDC25A (Fig. 6c). Thereafter, we determined KDM2B expression in CSCC cells and PCS480 cells as well. It was found that the mRNA and protein levels of KDM2B were increased in the CSCC cells (Fig. 6d-e). In addition, we also found a decline in H3K4me3 expression in the CDC25A promoter region of cells (Fig. 6f). To validate whether ALX3 recruits KDM2B to the promoter region of CDC25A, we first examined the sub-cellular localization of ALX3 and KDM2B in cells using double-labeled immunofluorescence staining. Importantly, both ALX3 and KDM2B were suggested to be localized in ME180 and C33A cells (Fig. 6g). In addition, a Co-IP assay was performed. An enrichment of KDM2B fragments was found in the compounds reacted by anti-ALX3 and accordingly, an enrichment of ALX3 fragments was found in the compounds pulled down by anti-KDM2B (Fig. 6h). These results, collectively, suggested that ALX3 recruits KDM2B to nuclei and promotes transcription of CDC25A.

### CDC25A activates the PI3K/Akt signaling pathway

**Fig. 7:**
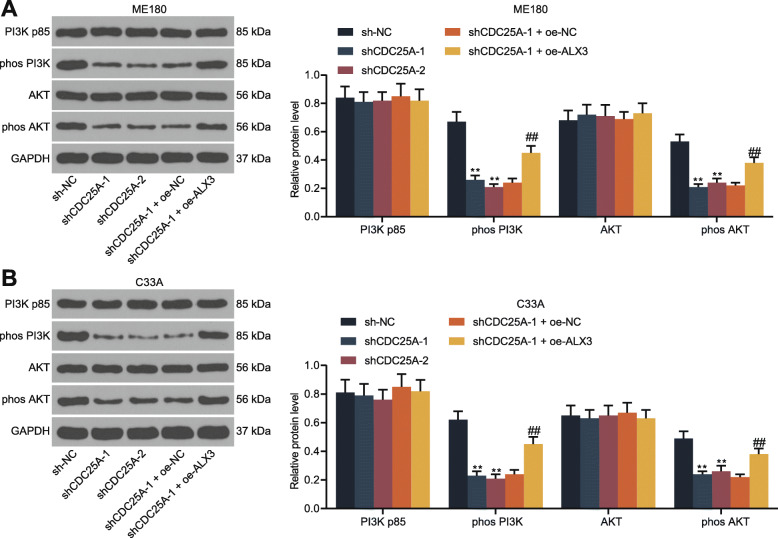
CDC25A activates the PI3K/Akt signaling pathway. **A**-**B**, phosphorylation of PI3K and AKT in ME180 (**A**) and C33A (**B**) cells determined by western blot analysis. Data were presented as the mean ± SD from three independent experiments. Data were analyzed by two-way ANOVA followed by Tukey’s multiple comparison test. ***p < 0.01 vs. sh-NC; ##*p* < 0.01 vs shCDC25A-1 + oe-NC

A previous report by Liu Y et al. suggested that upregulation of CDC25A led to activation of the PI3K/AKT signaling pathway to promote the malignant behaviors of glioma stem cells [21]. We therefore determined the phosphorylation level of PI3K and Akt in ME180 and C33A cells by western blot analysis. It was found that the phosphorylation of PI3K and Akt was significantly decreased following CDC25A downregulation but then recovered upon following ALX3 overexpression (Fig. 7a-b).

## Discussion

Expanding HPV vaccine usage is of primary importance, though, developing novel therapeutic options for the diagnosed patients is also an emergent issue for cervical cancer management. In the present study, we confirmed a novel regulatory network involving genetic and epigenetic regulations on CDC25A expression, where a transcription factor ALX3 recruits KDM2B to the promoter region of CDC25A which enhances CDC25A transcription through demethylation of H3K4me3, therefore leading to cell cycle progression and proliferation of cervical cancer cells.

Initially, the data on the GEO dataset GSE63514 suggested that CDC25A was highly expressed in CSCC samples and correlated with increased grading of tumors. A similar trend was obtained from the GEPIA database, where higher expression of CDC25A was predicted in the cervical cancer tissues. Experimentally, high expression of CDC25A was validated in the acquired CSCC cell lines. Further downregulation of CDC25A suppressed proliferation but induced cell cycle arrest at S phase in ME18 and C33A cells. The high-expression profile and the oncogenic role of CDC25A has been largely reported. For instance, downregulation of CDC25A reduced proliferation and cell cycle progression of liver cancer cells [22], breast cancer cells [23], and so forth. Moreover, CDC25A-mediated cell cycle progression has also been associated with chemo-resistance of cancer cells [24, 25]. In addition, in cervical cancer, CDC25A has been reported to reduce cell apoptosis whereas increase cell viability following radio therapy [26]. Also, CDC25A was identified as an important oncogene in cervical cancer during the transition between dysplasia and carcinoma [27]. In our study, in vitro results were reproduced in vivo, where downregulation of CDC25A reduced the growth rate of cervical cancer xenograft tumors in mice, which was partly in line with a previous study [28].

The subsequent bioinformatics analyses on the Ensembl, JASPAR and GEPIA databases suggested ALX3 was a transcription factor with a high correlation with CDC25A. The direct binding relationship between ALX3 and the CDC25A promoter was validated through ChIP-qPCR and luciferase reporter gene assays. Then, high expression of ALX3 was confirmed in the CSCC cell lines. ALX3 was reported as a DEG with a high DNA methylation in hepatocellular carcinoma [15]. Altered methylation of ALX3 was also found to be linked to colorectal cancer development [29]. Though the exact function of ALX3 on cancer has been rarely concerned, as a transcription factor, ALX3 has been reported to bind to the binding sites of foxo1 promoter, therefore scavenging oxidative stress and reducing the incidence of developmental malformations during diabetic gestations [30]. Here, our study confirmed that the above anti-proliferative events by CDC25A silencing were blocked upon ALX3 overexpression, indicating that ALX3 was at least partially responsible for CDC25A transcription and the cervical cancer progression.

Though initially less studied, the epigenetic mechanisms in cancer have aroused increasing focuses among researchers in this field [31]. In the present study, the data on the UCSC browser suggested that there are H3K4 sites on the CDC25A promoter region. Among the potential H3K4 mediators, KDM2B was predicted as a highly correlated demethylase with CDC25A according to the TCGA-CESC database. KDM2B is a ubiquitously expressed and conserved nuclear protein which targets H3K36me2 and H3K4me3 for demethylation and reported with multiple regulatory functions in cell senescence, proliferation and migration [32]. The oncogenic role of KDM2B has been reported in several human malignancies such as bladder cancer [33], breast cancer [34] and pancreatic cancer [35]. This is also true for gynecological cancers [34, 36], including cervical cancer [17]. Importantly, our following experiments suggested that the mRNA and protein expression of KDM2B was increased in the CSCC cell lines. Modification on H3K4me3 has shown either promoting or discouraging roles in gene expression. For instance, increased demethylation of H3K4me3 by KDM5C has been reported to inhibit the expression of LINC000231 that was related to the pathogenesis of cervical cancer [37]. In a study by Lv BB et al., increased methylation of H3K4me3 has been reported to be associated with the activation of the MDM2-p21-E2F1 axis [38]. On the other hand, KDM2B-mediated demethylation of H3K4me3, along with KDM6A-mediated demethylation of H3K27me3, has been found to promote EZH2 expression and the subsequent progression of non-small cell lung cancer [39]. Likewise, a similar KDM2B-H3K4me3-mediated EZH2 upregulation has been found in ovarian cancer cells [36]. Here in this paper, we confirmed that the H3K4me3 methylation in the CDC25A promoter was declined by KDM2B, which is possibly responsible for the upregulation of CDC25A mediated by ALX3. CDC25A has been reported as a positive regulator of the PI3K/Akt signaling pathway to promote the malignancy of glioma stem cells [21]. The PI3K/Akt signaling has been reported to be independently associated with CDC25A maintenance and the consequent cycle progression [40]. A traditional Chinese medicine Ganoderma tsugae has been observed to induce S phase arrest of lung adenocarcinoma cells by suppressing CDC25A and inactivating the PI3K/AKT signaling pathway [41]. Intriguingly, KDM2B was found to activate FAK and PI3K that mediate the motility of tumor cells [42]. The PI3K/Akt pathway is a frequently activated pathway implicated in cancer initiation and progression, therefore representing a key target for cancer treatment [43]. This is also applied in cervical cancer [44]. In our study, it was found that the phosphorylation of PI3K and Akt in cells was decreased following CDC25A knockdown but then recovered on ALX3 overexpression. These results indicated that activation of PI3K/Akt pathway was possibly implicated in the ALX3/KDM2B/CDC25A-mediated events.

## Conclusion

To conclude, this study demonstrated that ALX3 recruits KDM2B to promote expression of CDC25A through the demethylation of H3K4me3 in the promoter region of CDC25A, which consequently activates the PI3K/Akt pathway and promotes progression of CSCC. This study may offer novel insights into cervical cancer treatment. A major limitation of the study was that due to the limit in available clinical samples, clinical experiments were not involved in the present study. In addition, the exact mechanism by which CDC25A interacts with the PI3K/AKT activity remains unknown. We would like to investigate this issue and included clinical studies in our future researches. We also hope more studies will be conducted in the future to validate our findings and to provide more intensive understanding in the progression of cervical cancer.

## Supplementary Information

**Additional file 1: Supplementary** **Fig. S1.** Overexpression of CDC25A increases growth of PCS480 cells. A-B, mRNA (A) and protein (B) expression of CDC25A in PCS480 cells after CDC25A overexpression examined by RT-qPCR and western blot analysis, respectively; C-D, proliferation of PCS480 cells in the setting of CDC25A overexpression examined by EdU labeling (C) and colony formation (D) assays. Data were presented as mean ± SD from three independent experiments. In all panels, data were analyzed by the unpaired *t* test, **p < 0.01 vs. oe-NC. All cellular experiments were performed in triplicates and three duplicated wells were set.

**Additional file 2: Supplementary Fig. S2.** Overexpression of ALX3 increases growth of PCS480 cells. A-B, mRNA (A) and protein (B) expression of ALX3 and CDC25A in PCS480 cells after ALX3 overexpression examined by RT-qPCR and western blot analysis, respectively; C-D, proliferation of PCS480 cells in the setting of ALX3 overexpression examined by EdU labeling (C) and colony formation (D) assays. Data were presented as mean ± SD from three independent experiments. In panels A and B, data were analyzed by two-way ANOVA and Tukey’s multiple comparison test; in panels C and D, data were analyzed by the unpaired t test, **p < 0.01 vs. oe-NC. All cellular experiments were performed in triplicates and three duplicated wells were set.

**Additional file 3.** Original images of protein bands.

## Data Availability

All the data generated or analyzed during this study are included in this published article. The GSE63514 dataset is available at (https://www.ncbi.nlm.nih.gov/geo/query/acc.cgi?acc=GSE63514).

## Abbreviations

ANOVA: Analysis of variance
BCA: Bicinchoninic acid
BSA: Bovine serum albumin
CDC25A: Cell division cycle 25A
ChIP: Chromatin immunoprecipitation
CSCC: Cervical squamous cell carcinoma
CST: Cell Signaling Technologies
DAPI: 4′, 6-diamidino-2-phenylindole
DMEM: Dulbecco’s modified Eagle’s medium
EdU: 5-ethynyl-2′-deoxyuridine
FITC: Fluorescein isothiocyanate
GEPIA: Gene Expression Profiling Interactive Analysis
H3K4me3: H3 trimethylation at lysine 4
HPV: Human papillomavirus
IHC staining: Immunohistochemical staining
KDM2B: Lysine demethylase 2B
mean ± SD: mean ± standard deviation
PBS: Phosphate-buffered saline
PI: Propidium iodide
RT-qPCR: Reverse transcription quantitative polymerase chain reaction
shRNAs: short hairpin RNAs
TCGA: The Cancer Genome Atlas
TUNEL: Terminal deoxynucleotidyl transferase (TdT)-mediated dUTP nick end labeling
