# Supplementary material for: Oncogenic role of ALX3 in cervical cancer cells through KDM2B-mediated histone demethylation of CDC25A
Source: BMC Cancer. 2021 Jul 16;21:819. doi: 10.1186/s12885-021-08552-7 (PMC8284019; doi:10.1186/s12885-021-08552-7)

Fig. 1F

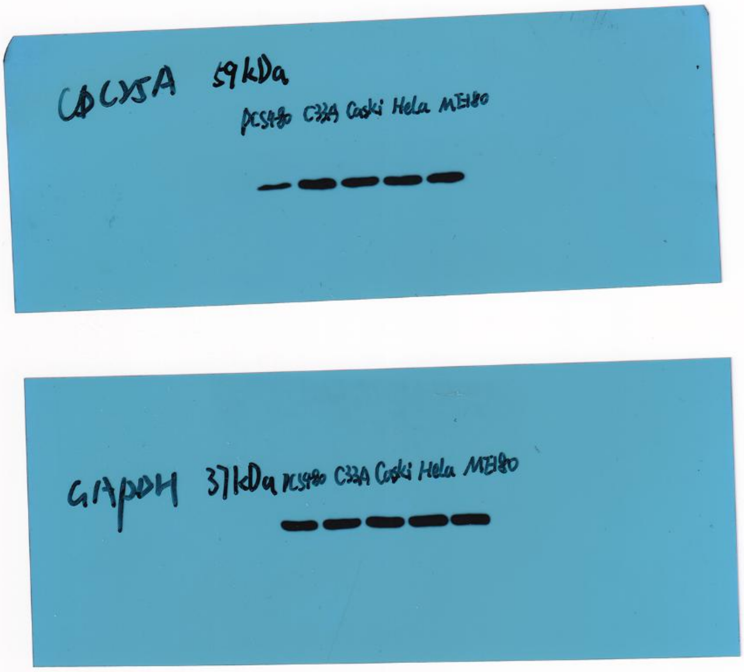

59kDa
PCS480 C33A Caski Hela ME180
GAPDH 37kDa PCS480 C33A Caski Hela ME180

Fig. 2B

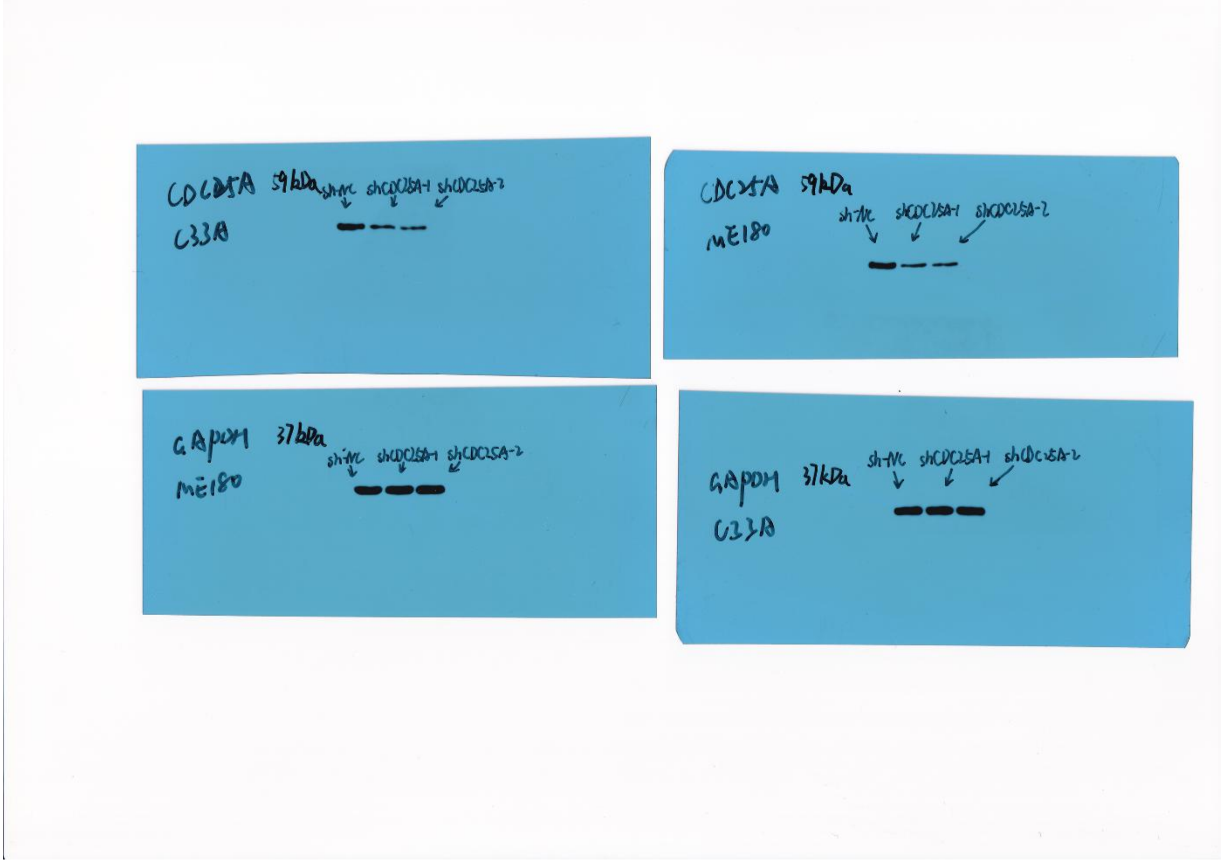
CDC25A 59kDa
C33A
sh-NC
shCDC25A-1
shCDC25A-2
CDC25A 59kDa
ME180
GAPDH 37kDa
ME180
GAPDH 37kDa
C33A

Fig. 2E

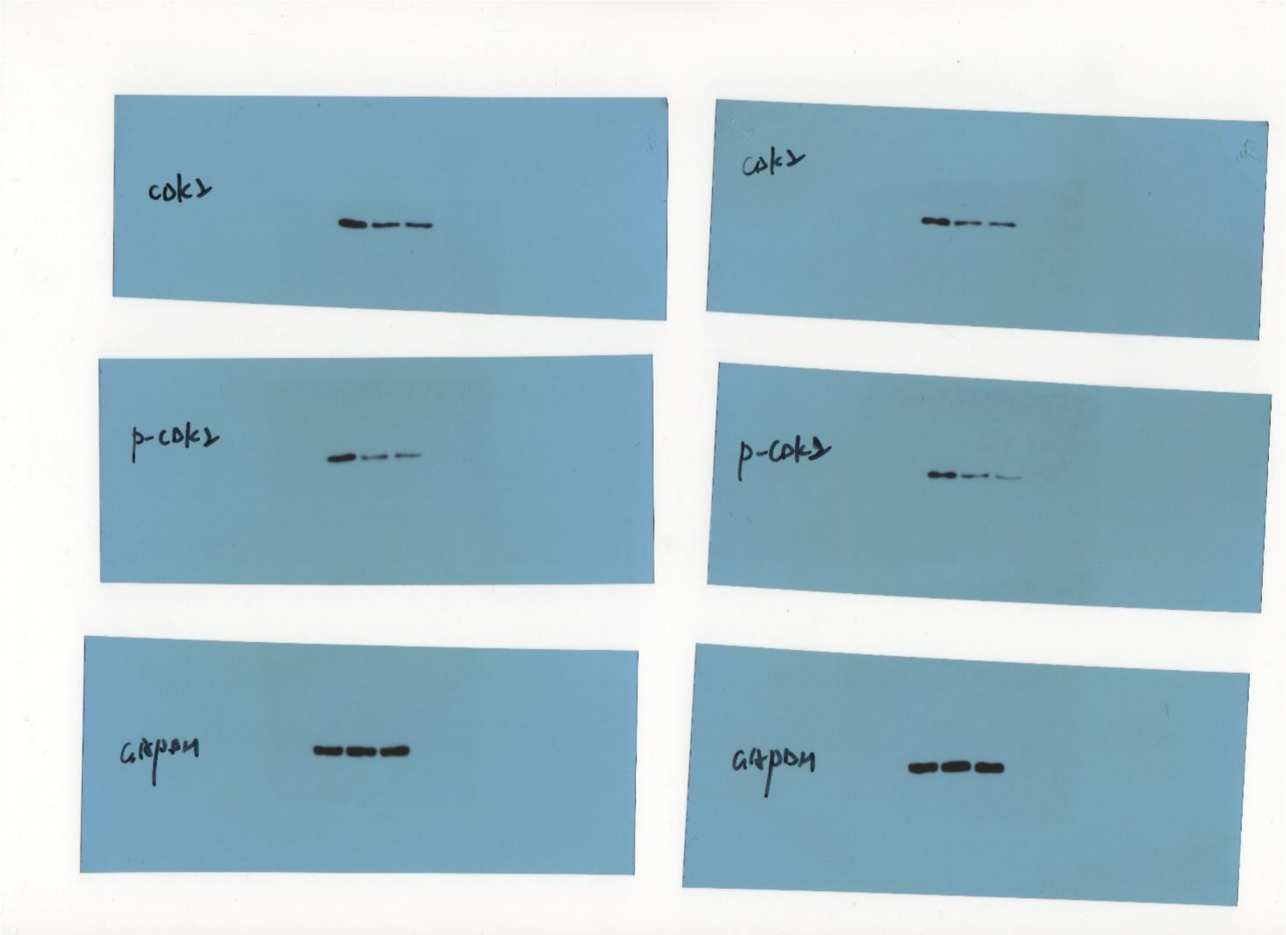

Fig. 4I

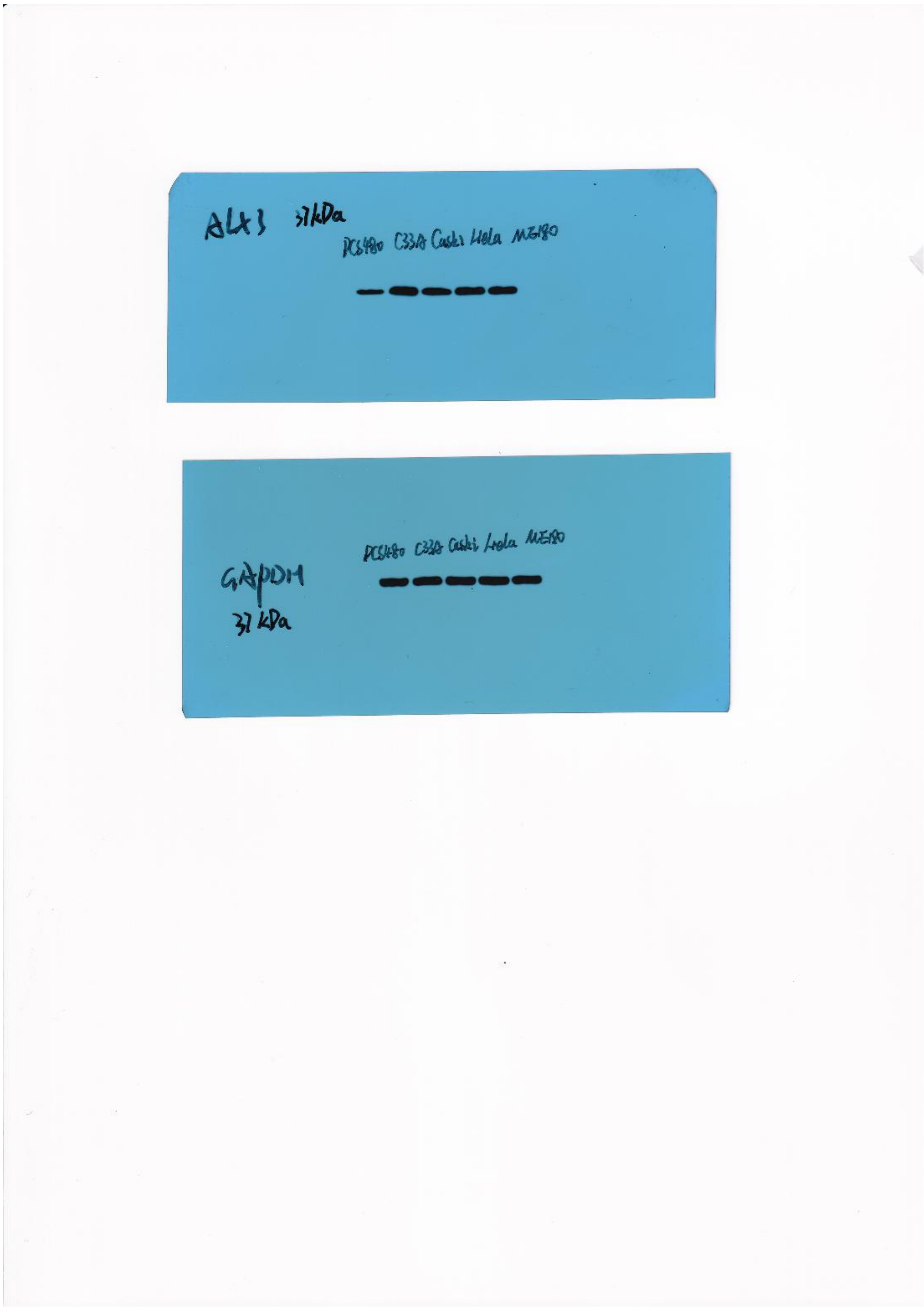

Fig. 4K

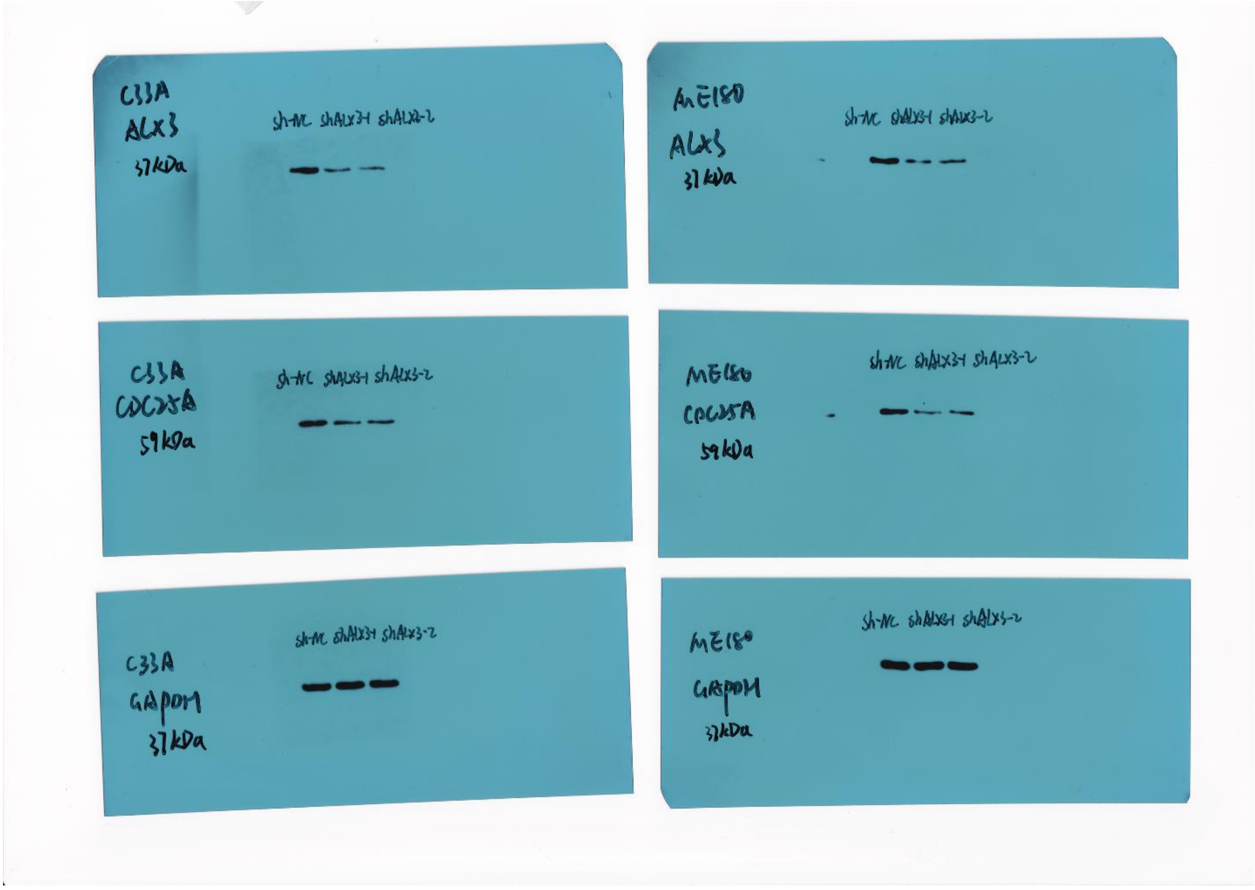
C33A
ALX3
37kDa
sh-NC shALX3-1 shALX3-2
ME180
ALX3
37kDa
sh-NC shALX3-1 shALX3-2
C33A
CDC25A
59kDa
sh-NC shALX3-1 shALX3-2
ME180
CDC25A
59kDa
sh-NC shALX3-1 shALX3-2
C33A
GAPDH
37kDa
sh-NC shALX3-1 shALX3-2
ME180
GAPDH
37kDa
sh-NC shALX3-1 shALX3-2

Fig. 5B

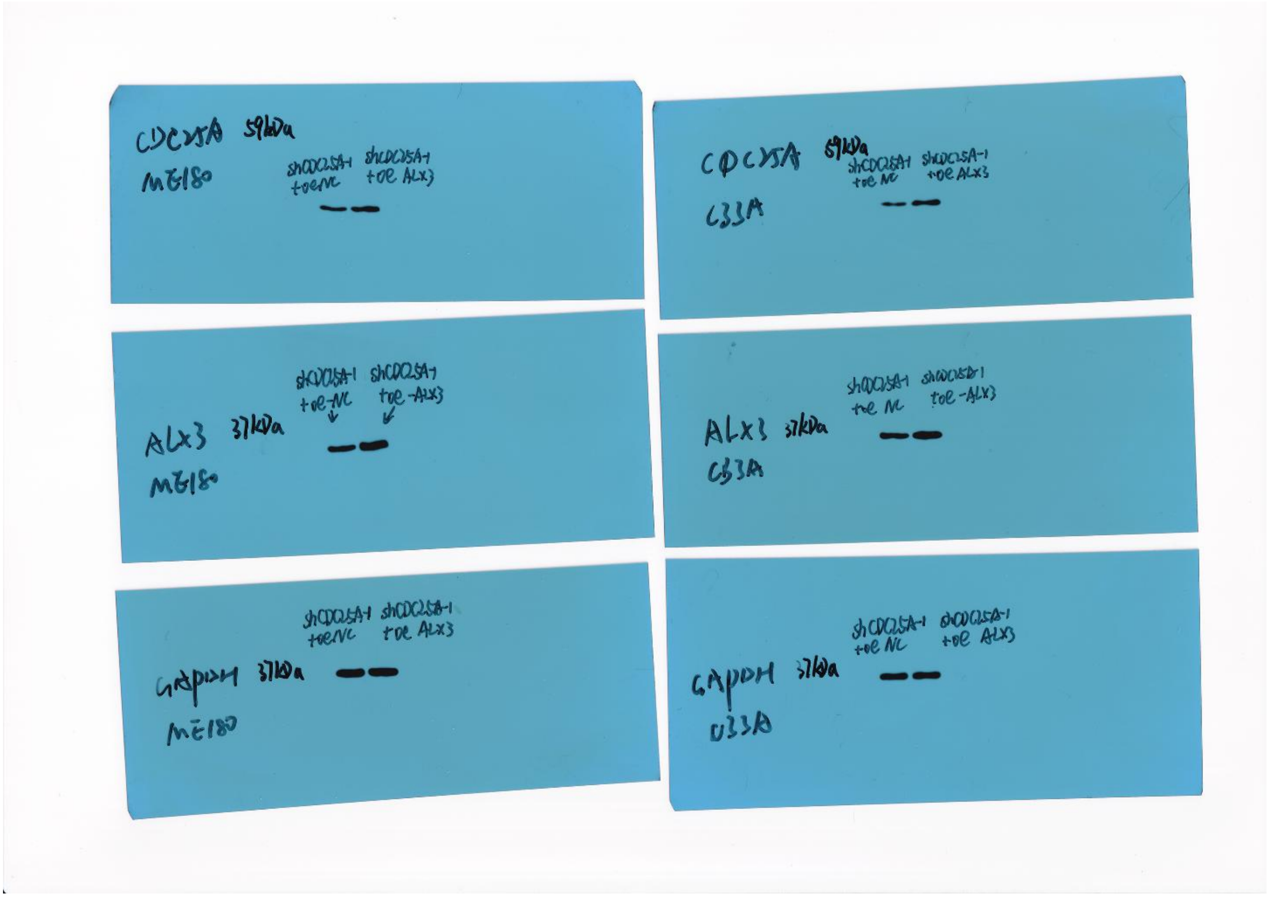
CDC25A 59kDa
ME180
shCDC25A-1 +oe NC
shCDC25A-1 +oe ALX3
CDC25A 59kDa
C33A
ALX3 37kDa
ME180
ALX3 37kDa
C33A
GAPDH 37kDa
ME180
GAPDH 37kDa
C33A

Fig. 6B

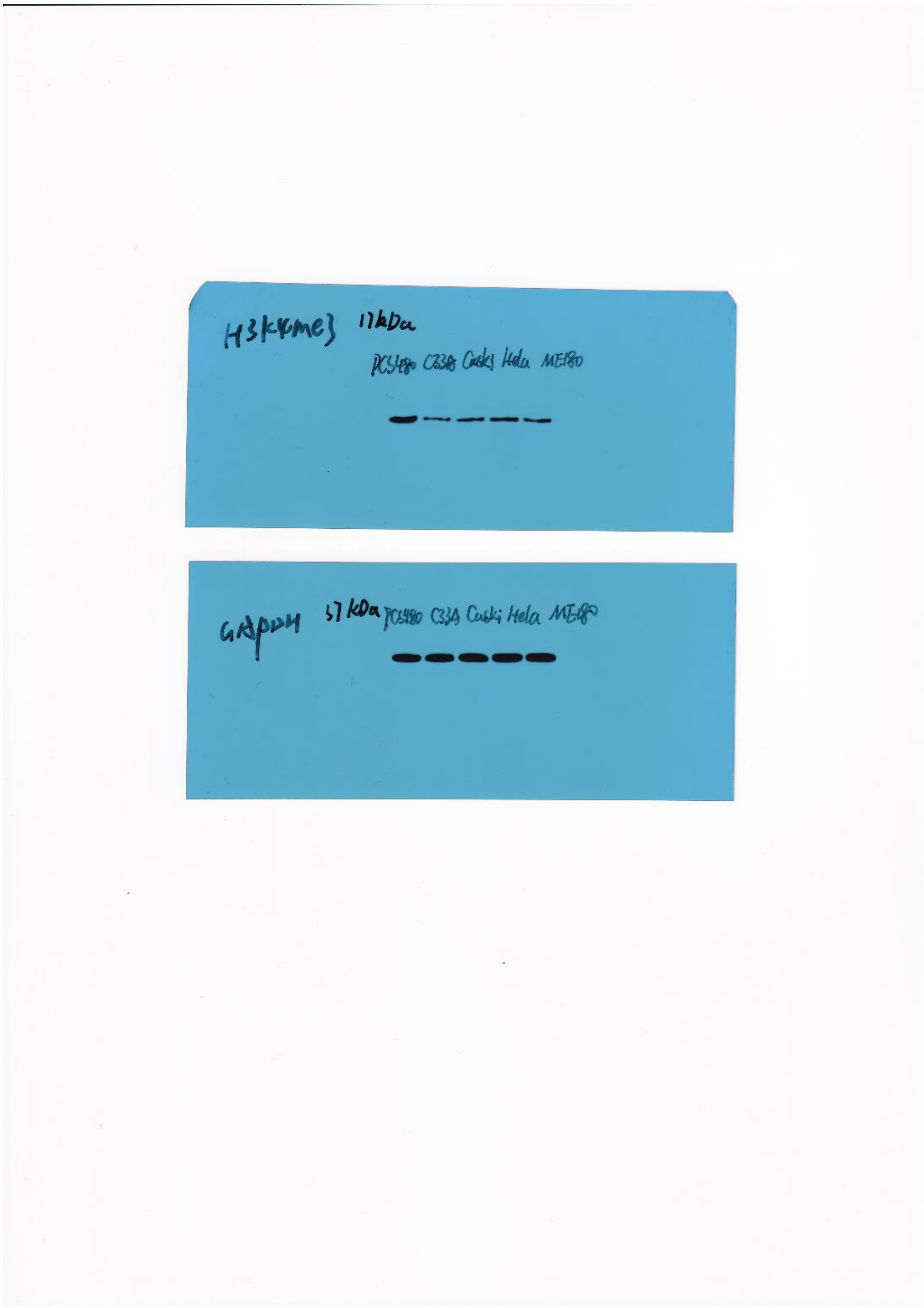

Fig. 6E

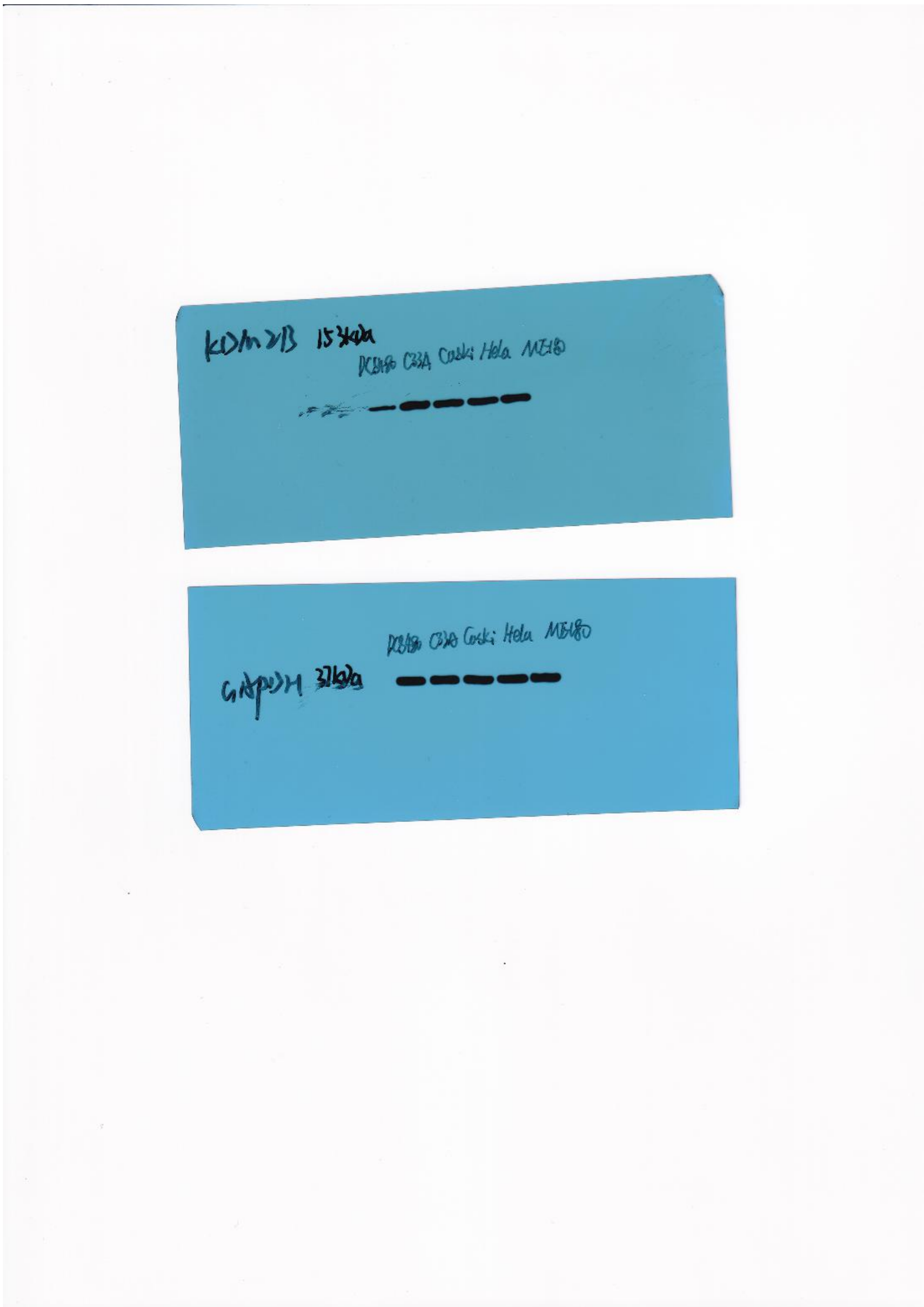

Fig. 6H

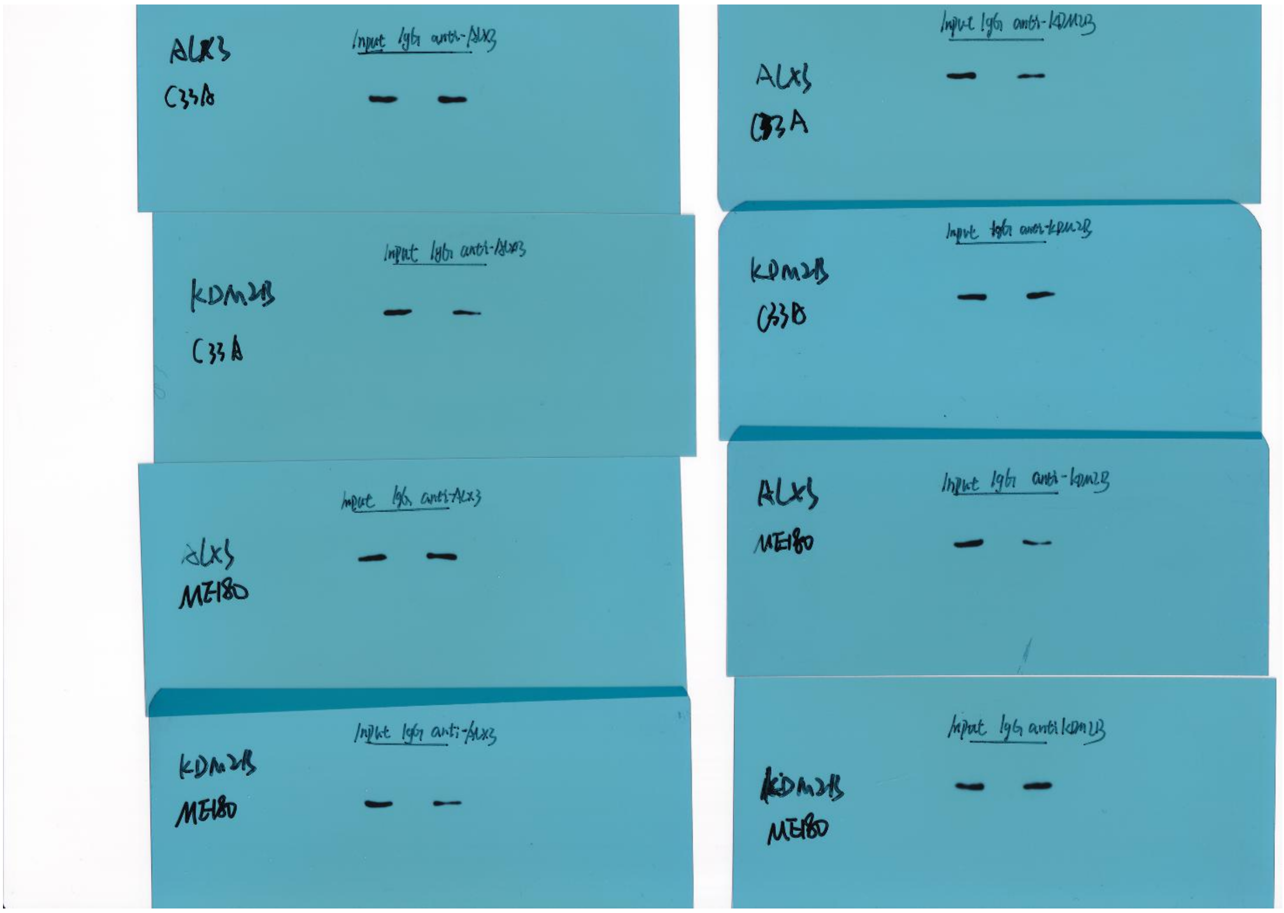
ALX3
C33A
Input IgG anti-ALX3
KDM2B
C33A
Input IgG anti-ALX3
ALX3
ME180
Input IgG anti-ALX3
KDM2B
ME180
Input IgG anti-ALX3
ALX3
C33A
Input IgG anti-KDM2B
KDM2B
C33A
Input IgG anti-KDM2B
ALX3
ME180
Input IgG anti-KDM2B
KDM2B
ME180
Input IgG anti-KDM2B

Fig. 7A

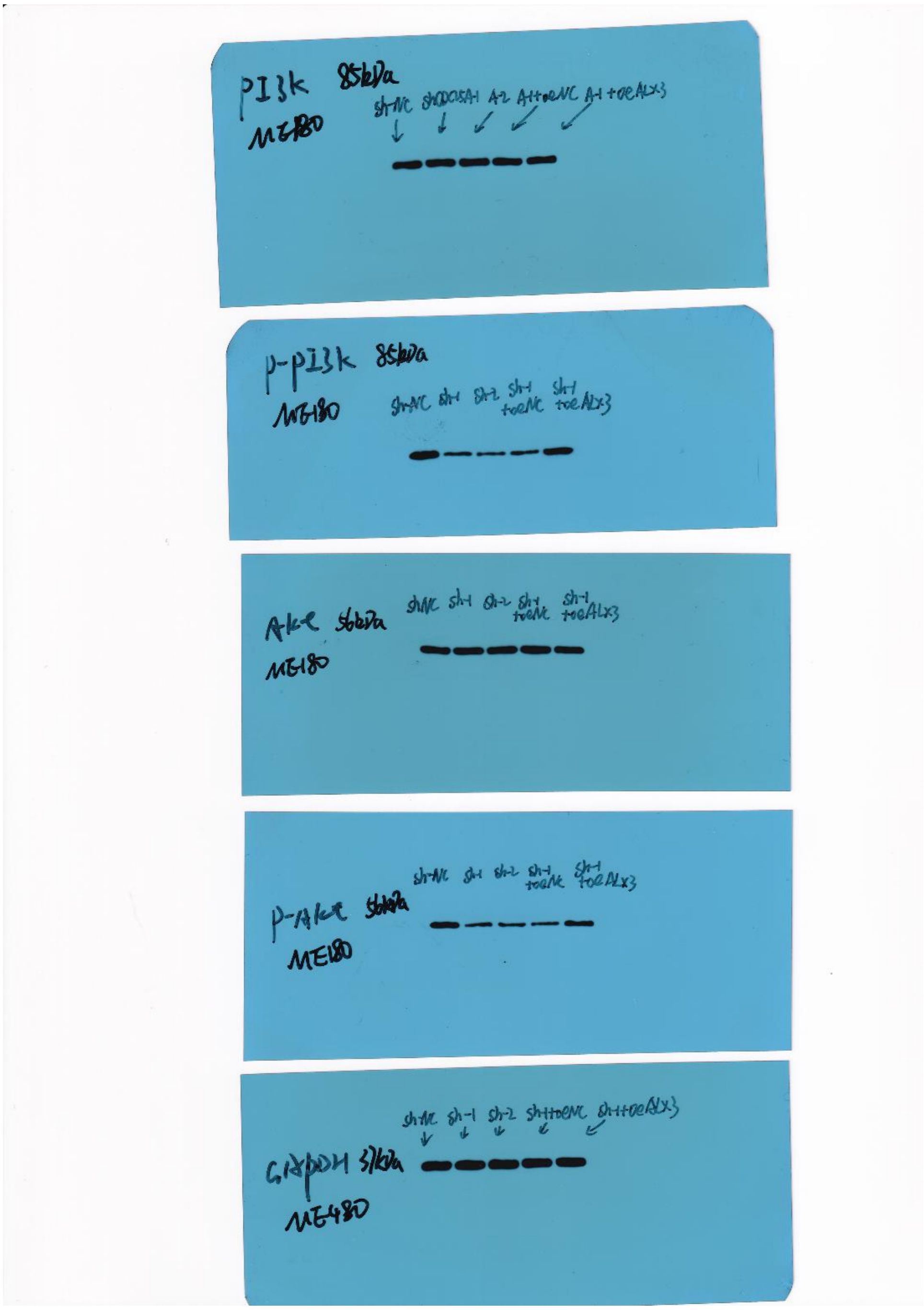
PI3k 85kDa
ME180
p-PI3k 85kDa
ME180
Akt 56kDa
ME180
p-Akt 56kDa
ME180
GAPDH 37kDa
ME480

Fig. 7B

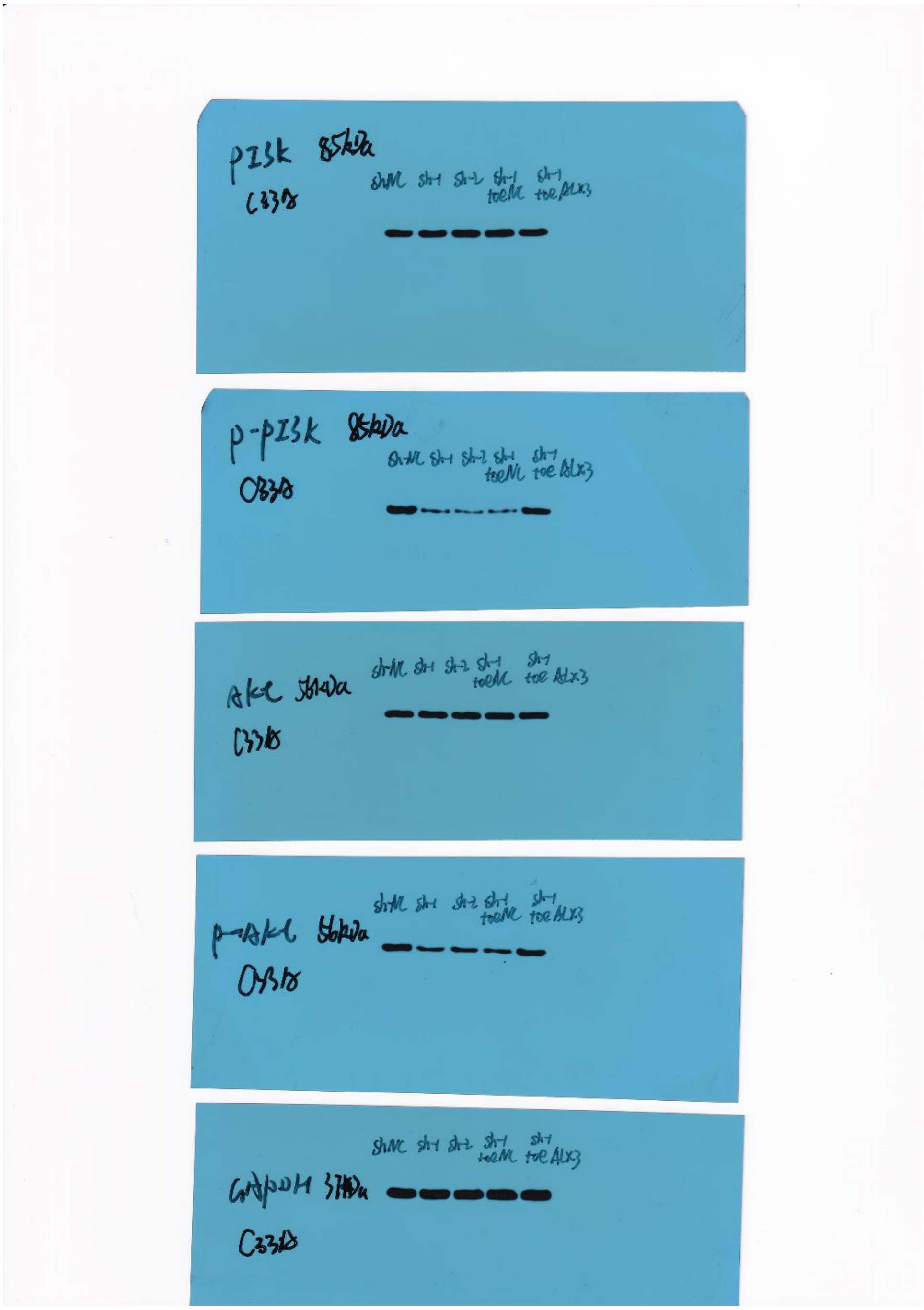

Fig. S1B

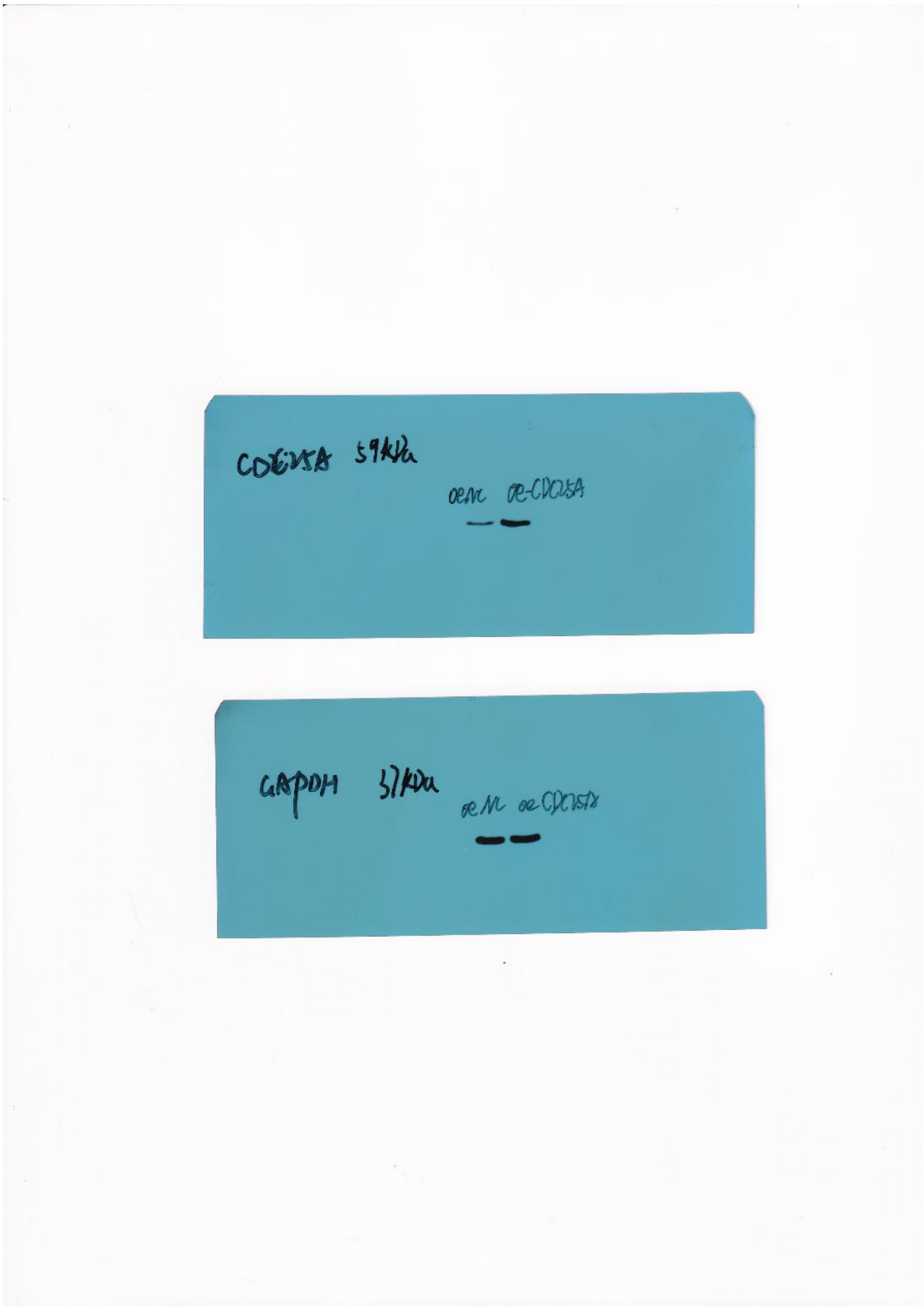

Fig. S2B

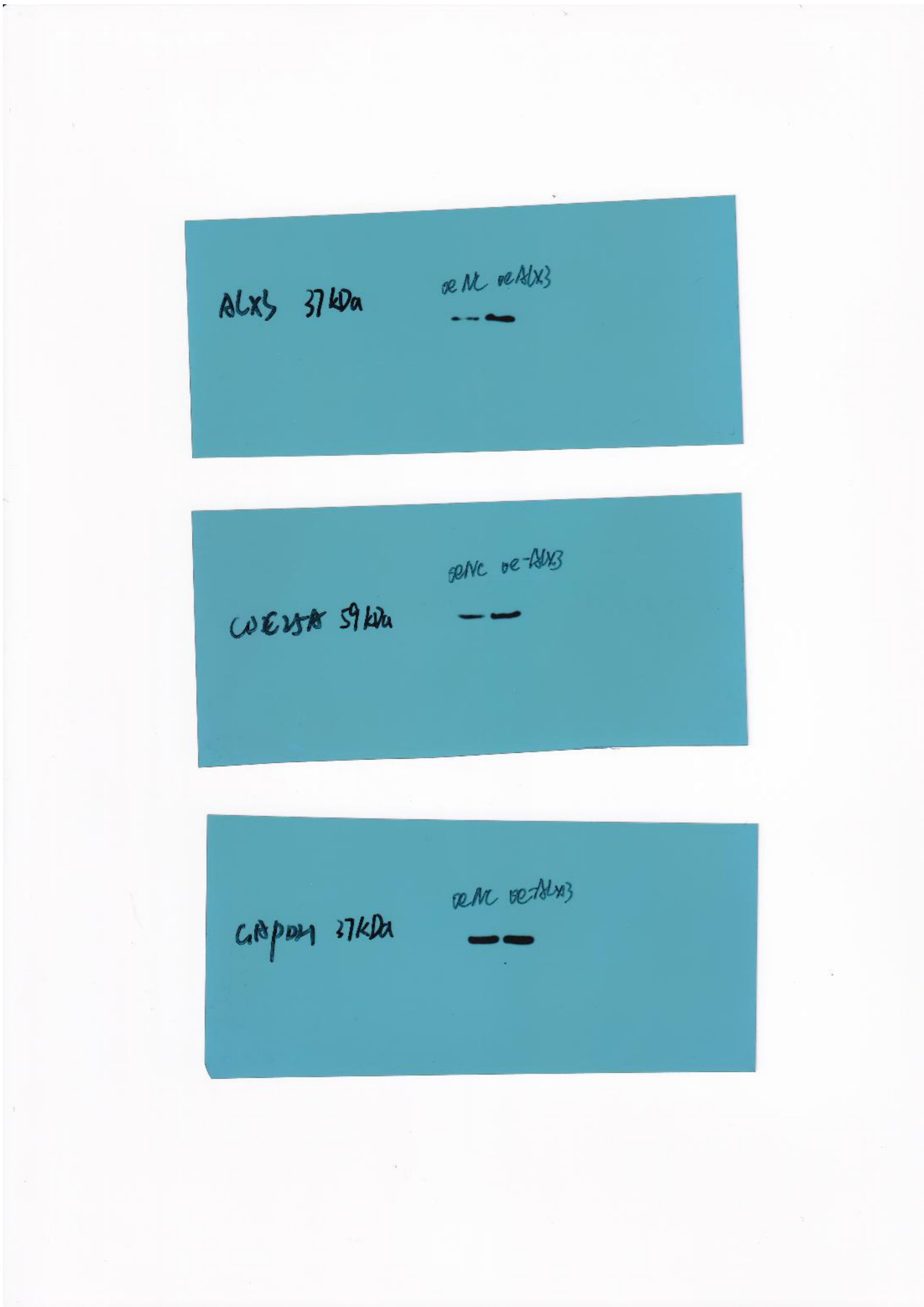

Supplement: Supplementary file 3 — Additional file 3. Original images of protein bands. [file 12885_2021_8552_MOESM3_ESM.pdf]
